# Low intensity extracorporeal shockwave Therapy shifts PDE5i nonresponders to responders

**DOI:** 10.1590/S1677-5538.IBJU.2019.0374

**Published:** 2020-09-02

**Authors:** Jiamin Wang, Lianmin Luo, Shankun Zhao, Yangzhou Liu, Zhiguo Zhu, Zhigang Zhao

**Affiliations:** 1 Guangdong Provincial Key Laboratory of Urology The First Affiliated Hospital GuangZhou Medical University GuangzhouGuangdong China Department of Urology & Andrology, Minimally Invasive Surgery Center, Guangdong Provincial Key Laboratory of Urology, The First Affiliated Hospital of GuangZhou Medical University, Guangzhou, Guangdong, China

**Keywords:** Erectile Dysfunction, Extracorporeal Shockwave Therapy, Pharmaceutical Preparations

## Abstract

To evaluate the efficiency of an energy density of 0.05mj/mm^2^ of low intensity extracorporeal shockwave therapy (Li-ESWT) on erectile dysfunction (ED) patients.A total of 45 ED patients met the inclusion criteria, including 7 PDE5i responders and 38 nonresponders. All the patients have already been delivered 10000 shockwaves of total seven treatment points twice a week for 4 weeks. Simultaneously, questionnaires of International Index of Erectile Function-Erectile Function (IIEF-EF), Erectile Hard Score (EHS) and Minimal Clinical Important Differences (MCID) were evaluated for the efficiency and safety at 8^th^ and 16^th^ weeks.The changes in the IIEF-EF score by MCID suggested that Li-ESWT treatment was effective in 22 PDE5i nonresponders patients (58%) at 8^th^ week. Then at 16^th^ week the number of patients who were effectively treated increased to 27 (71%). Among PDE5i responders, 5 patients (71%) were effective base on MCID at 16^th^ week. Among PDE5i nonresponders 22 patients (58%) achieved erection hard enough for vaginal penetration and increased to 27 (71%) patients at 16^th^ week (EHS ≥3). Moreover, even 3 patients achieved EHS 4 in PDE5i nonresponders at 16^th^ week. Among PDE5i responders, 4 of 7 patients reached EHS of 4 from EHS 3 at 16^th^ week. Apart from this, Li-ESWT treatment was also effective in 9 patients (24%) in PDE5i nonresponders without follow-up PDE5i.Energy flux density (EFD) of 0.05 of Li-ESWT could improve the erectile function of ED patients with PDE5i response. In addition, EFD of 0.05 of Li-ESWT treatment could turn PDE5i nonresponders to responders.

## INTRODUCTION

Erectile dysfunction (ED) is a male sexual dysfunction defined as a consistent or recurrent inability to attain or maintain an erection sufficient for sexual intercourse ( 1 ). The current mainstream treatments are the use of oral phosphodiesterase type 5 inhibitor (PDE5i), low intensity extracorporeal shockwave therapy (Li-ESWT), intracavernous injections of vasodilating agents and penile prostheses ( 2 ). None of them can improve the underlying pathophysiological changes of erectile dysfunction, except for Li-ESWT ( 3 ). However, up to 35% of ED patients do not respond with PDE5i and are prone to the most common side effects such as headaches and blushing ( 4 ). Patients using PDE-5i should be also warned about a possible link between PDE-5i use and occurrence of hearing impairment ( 5 ). Intracavernous injections are effective but their use requires careful dose titration and some precautions. Complications of intracavernous drugs include pain, priapism and corpora cavernosa fibrosis ( 4 ). Penile prostheses will be considered to treat severe ED when all conservative treatments have failed. The treatment of PDE-5i for ED enhance sexual function by improving the quality of single erections. So it is quite significant to found a supplement therapy to patients.

A novel method to prevent the deterioration of erectile function due to these pathophysiologic processes is desperately needed. Animal studies have demonstrated neoangiogenesis in myocardial tissue and skin flaps, which invites the hypothesis that erectile dysfunction of vascular origin could be treated by Li-ESWT ( 6 - 8 ). Previous studies have shown that focal Li-ESWT can have a positive effect in men with ED ( 9 - 13 ). Li-ESWT inducts cellular microtrauma, and promotes angiogenesis by enhancing the expression of vascular endothelial growth factor and recruitment of endothelial progenitor cells ( 14 ).

Although Li-ESWT was reported to be effective, minimally invasive or non-invasive in the treatment of ED patients, it is not a fully-fledged, accredited treatment program. Nevertheless, almost all clinical trials used an energy flux density (EFD) of 0.09mj/mm^2^ of shockwaves after Vardi et al. ( 15 ) first reported in 2010. However, evidences on this area are still scarce. The therapeutic parameter, like EFD, has not been a unanimous agreement yet. Several articles reported the feasibility of other EFDs of Li-ESWT in animal model, like 0.05mj/mm^2^ ( 16 - 19 ).

Li et al. ( 16 ) reported that EFD of 0.05mj/mm^2^ of Li-ESWT could significantly improve pelvic neurovascular injury by bilateral cavernous nerve injury and internal pudendal bundle injury (PVNI) impaired erectile function, enhancing penile angiogenesis and regain of blood circulation in PVNI rat model. EFD of 0.05mj/mm^2^ of Li-ESWT could activate local penile progenitor cells of rats and contribute to the beneficial effects of shockwave treatment for erectile dysfunction, which represents a non-invasive alternative to exogenous stem cell therapy ( 18 ).

Thus, we investigated and monitored the treatment of different EFD (0.05mj/mm^2^) of Li-ESWT for ED. To our knowledge this is the first clinical study that evaluated efficacy and safety after an energy density of 0.05mj/mm^2^ of Li-ESWT in men with ED.

## MATERIALS AND METHODS

The study protocol was reviewed and approved by The First Affiliated Hospital of GuangZhou Medical University. Guangdong, China, Ethics Committee (YKLS2017NO.26). The study is listed in Chinese Clinical Trial Registry (ChiCTR-IIR-17011554). An independent clinical research unit monitored the research process.

### Screening, Inclusion and Exclusion Criteria

Patients complaining of ED during a consultation at our outpatient clinic for other indications also were offered participation in the trial. Inclusion criteria included: 1) Accord with ED; 2) Stable heterosexual relationships at least 3 months before treatment. Exclusion criteria were: 1) Patients who had radical prostatectomy or pelvic surgery before; 2) Any condition that may not be compatible with the completion of treatment, as judged by a doctor, such as an unstable mental condition that is not controlled by drugs, spinal cord injury, penile anatomical abnormality, excessive obesity; 3) Patients with penile prosthesis; 4) Patients who have recovered from cancer in the past year or who have serious illnesses of vital organs; 5) Serious hematomas; 6) Venous leakage, diagnosed by cavernosography; 7) Anti-androgen, oral or injected androgen; 8) After pelvic radiotherapy; 9) Coagulation dysfunction or the use of anticoagulants (such as coumarin); 10) Participated in any other medical device or drug clinical investigator in the past three months; 11) Other patients who were considered not suitable in this trial.

### Study Protocol

**Figure 1:**
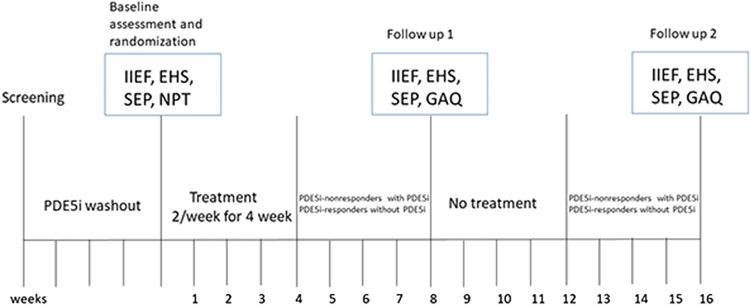
Study flowchart.

When subjects met the inclusion criteria and returned a signed consent form, they could enter the trial. All subjects consented not to use additional erectile dysfunction treatment outside this study protocol during the treatment. They who previously have used PDE5i should underwent a 4-week washout period before the Li-ESWT treatment. Each study patient had abnormal 2-night Nocturnal Penile Tumescence (NPT) parameters at screening. Serum glucose, lipid profile, and total testosterone level as well as several validated sexual function questionnaires including International Index of Erectile Function-Erectile Function (IIEF-EF), Sexual Encounter Profile (SEP), Erection Hardness Score (EHS), were assessed at baseline. These indexes except NPT and the questionnaire Global Assessment Questions (GAQ) were performed again at 8^th^ week. Finally only the questionnaires (IIEF-EF, EHS, GAQ, and SEP) were assessed at 16^th^ week. PDE5i nonresponders received 25mg of sildenafil after Li-ESWT treatment and before assessment at 8^th^ week and 16^th^ week. Details are listed in Figure-1 . Subjects completed the questionnaires using tables in a separate room and were not disturbed by other participants or investigators.

### Li-ESWT Specifications

Shockwaves were applied to the corporal cavernosa using a focused shockwave source with lower energy density of 0.05mj/mm^2^ (HB-ESWT-01, Zhanjiang Haibin Medical Equipment Co., Ltd, Zhanjiang, Guangdong, China). We applied a standard commercial gel normally used for sonography without any local anesthetic effect on the penis and perineum, and the penis was stretched manually. All patients were treated twice a week and the treatment course was 4 weeks, comprising of 10.000 shockwaves delivered to seven treatment points at two sides of the distal and proximal penile shaft (1600 shockwaves to each position), and corporal bodies on the perineum (1200 shockwaves to each crura and in between). The 10.000 shocks for 7 foci at an energy density of 0.05mJ/mm^2^ and a frequency of 200/min were delivered according to the manufacturer’s guidelines. Each treatment session lasted 50 min. The shockwave generator implemented in our study has been used in the treatment of tenosynovitis and tendonitis. During the treatment period, patients were required to maintain their normal sexual habits if they could. PDE5i nonresponders who were prescribed sildenafil were observed after treatment of 4 weeks of as-needed, flexible-dose treatment with sildenafil citrate at the recommended initial dose of 25mg.

### Main Outcome Measures

The primary outcome measurement was made at the 8^th^ and 16^th^weeks, which represented 4 and 12 weeks after completion of treatment, change from baseline for IIEF-EF score. Secondary outcome measures included the interval change of EHS, SEP and GAQ, as well as adverse events from Li-ESWT therapy. Treatment success was defined as EHS 3 or greater, which indicated that the erectile function was hard enough for vaginal penetration. Furthermore, we evaluated an improvement on IIEF-EF according to Minimal Clinical Important Differences (MCID) criteria, at least 4 points improvement for ED patients ( 20 ).

### Statistical Analysis

SPSS 20.0 software (SPSS, Chicago, IL, USA) was used to analyze the data. The results were expressed as median (interquartile range IQR). The values of the study variables were compared using the Student t-test or Wilcoxon signed-rank test, as appropriate. A Fisher’s exact test was applied to measure the levels of EHS or the questionnaires GAQ and SEP. The level of significance for all analyses was p <0.05.

## RESULTS

45 patients were recruited into this trial, including 7 PDE5i responders and 38 nonresponders patients. Table-1 lists patient baseline parameters. Participants were mostly middle-aged men with long-lasting severe ED. Treatment success of PDE5i responders and nonresponders is listed in Table-2 . At 8^th^ week follow-up examination the median IIEF-EF score increased from 10 (IQR 8-11) at baseline to 15 (IQR 12-16) in PDE5i nonresponders group and from 16 (IQR 16-18) to 21 (IQR 20-22) in PDE5i responders. Moreover, at 16^th^ week, the score increased to 16 (IQR 14-17) in PDE5i responders and still 21 (IQR 20-23) in PDE5i nonresponders.

**Table 1:** Baseline characteristics of study population.

	Li-ESWT	

	PDE5i responders	PDE5i-nonresponders
Participants (n)	7	38
Median age (range)	60 (30-81)	61 (30-84)
Median Duration of ED (months)	33 ( 12 -60)	43 ( 6 -120)
Incidence of ED risk factors (n)	6	32
Diabetes	1	2
Hypertension	1	1
Ischemic heart disease	0	0
Smoker	4	29
Disease stratification*		
Mild/mild to moderate	7	9
Moderate	0	23
Severe	0	6
Median baseline IIEF-5 score	16 ( 16 - 18 )	10 ( 8 - 11 )

* = Mild/mild to moderate: 12-21; moderate: 8-11; severe: 5-7.

**Table 2:** Treatment success of LI-ESWT.

	Li-ESWT	

	PDE5i responders	PDE5i-nonresponders
NO. patients	7	38
**Median IIEF-5**		
baseline	16 ( 16 - 18 )	10 ( 8 - 11 )
after treatment(8th week)	21 ( 20 - 22 )***	15 ( 12 - 16 )***
after treatment(16th week)	21 ( 20 -23)***	16 ( 14 - 17 )***
△after treatment(8th week)	5 ( 1 - 6 )***	5 ( 4 - 6 )***
△after treatment(16th week)	6 ( 1 - 6 )***	6 ( 4 - 7 )***
**Success (%)(8th week)**		
IIEF-5(MCID)	4 (57%)***	22 (58%)***
△EHS ≥ 3	4 (57%)***	22 (58%)***
**Success (%)(16th week)**		
IIEF-5(MCID)	5 (71%)***	27 (71%)***
without PDE5i		9 (24%)
△EHS ≥ 3	4 (57%)***	27 (71%)***
Both GAQ1&2 are "YES" (16th week)	5 (71%)***	28 (74%)***
Both SEP1&2 are "YES" (16th week)	4 (57%)***	25 (66%)***

*** = A significant improvement was found at Li-ESWT group vs sham or baseline (P=0.000).

△ = A change from baseline.

**Figure 2:**
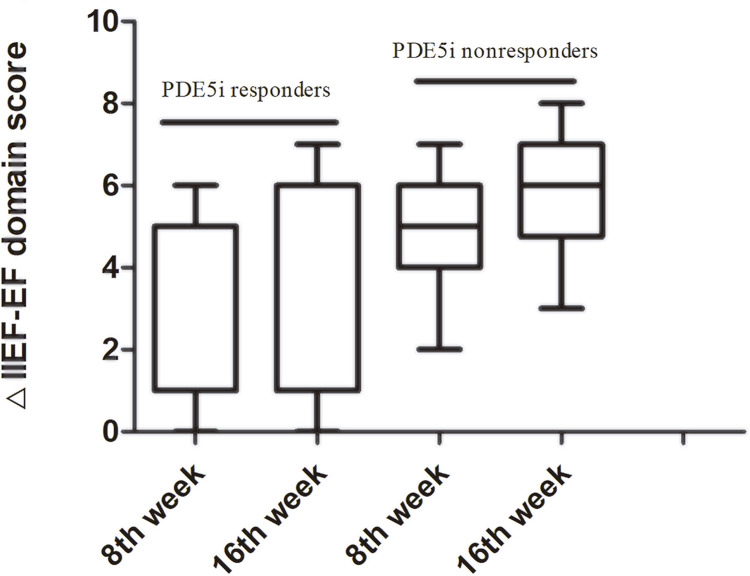
△ A change from baseline. PDE5i nonresponders will have PDE5i before assessment both in 8 **th** and 16 **th** week, but PDE5i responders not.

The median change after treatment in IIEF-EF score in the PDE5i responders and nonresponders were 5 (IQR 4-6), 6 (IQR 4-7) (P=0.005, all) and 5 (IQR 1-6), 6 (IQR 1-6) (P=0.000, all) at 8^th^ week, and 16^th^week, respectively ( Table-2 and Figure-2 ). According to the changes in the IIEF-EF score by MCID the treatment was effective in 22 PDE5i nonresponders patients (58%) at 8^th^week and increased to 27 patients (71%) at 16^th^ week. In PDE5i responders, 5 patients (71%) were effective based on MCID at 16^th^week ( Table-2 ).

**Figure 3:**
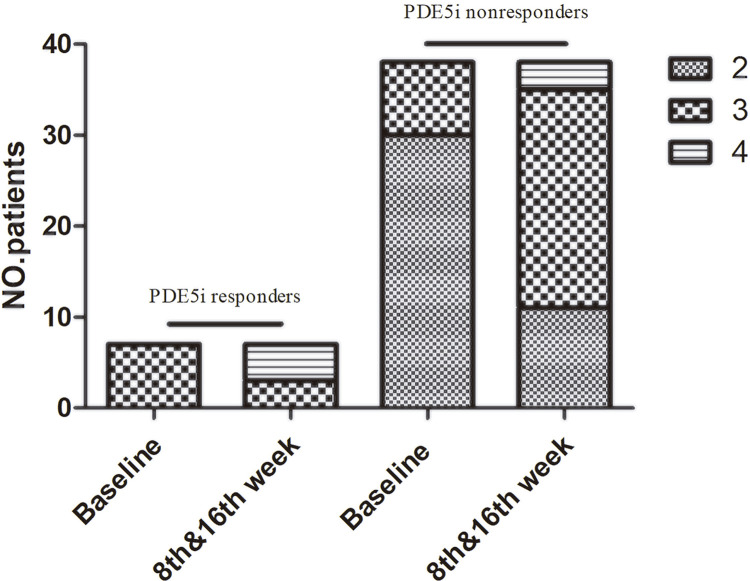
PDE5i nonresponders will have PDE5i before assessment both in 8 **th** and 16 **th** week, but PDE5i responders not.

In PDE5i nonresponders 22 patients (58%) achieved erection hard enough for vaginal penetration and increased to 27 (71%) patients at 16^th^week (EHS ≥3, P=0.000, Table-2 and Figure-3 ). And even 3 patients achieved EHS 4 in PDE5i nonresponders at 16^th^ week. As to PDE5i responders, 4 of 7 patients achieved to EHS of 4 from EHS of 3 at 16^th^ week ( Table-2 and Figure-3 ). 9 patients (24%) in PDE5i nonresponders achieved EHS ≥3 without PDE5i after Li-ESWT treatment ( Table-2 ). At 16^th^ week, 28 patients (74%) PDE5i nonresponders marked “Yes” in both GAQ 1 & 2 and 25 patients (66%) marked “Yes” in both SEP 2 & 3 vs. 4 or 5 of 7 patients in PDE5i responders in GAQ & SEP, respectively ( Table-2 ). No adverse event, like pain, hematoma, hematuria and bruising, was reported during treatment and after the intervention.

## DISCUSSION

Several clinical trials conducted by different institutions have supported the effectiveness of Li-ESWT to ED patients, since Vardi et al. ( 15 ) first discovered that in 2010. However, the success rate of the EFD of 0.09mj/mm^2^ of Li-ESWT treatment for ED patients who do not respond to PDE5i is largely unsuccessfully ( 10 ). Data on the setup parameters or treatment protocols of Li-ESWT to restore spontaneous erectile function are still scarce. Majority of the studies simply follow the protocol base on Vardi et al. ( 15 ). To identify potential possible positive responses, we should explore different treatment options for Li-ESWT, including energy density, treatment sites, shockwaves, etc.

Based on this present study, about 70% PDE5i nonresponders patients who were treated with Li-ESWT at first step were able to achieved erection hard enough for vaginal penetration after using PDE5i at second step, and it lasted 3 months at least. Li-ESWT was able to convert true PDE5i nonresponders to responders. PDE5i nonresponders became drug-sensitive after Li-ESWT treatment. About 70% patients treated with PDE5i after Li-ESWT achieved good enough erection to have intercourse. Notably 7 patients of all Li-ESWT group achieved full erection (EHS=4) with or without PDE5i. In PDE5i responders, though the sample size is small, 70% of them were able to have intercourse without medication after Li-ESWT treatment. The improvement of erectile function was found in from 8^th^ week to 16^th^ week, which implied that the potential pathological changes was improved. Moreover 9 of PDE5i nonresponders patients achieved erection strong enough for vaginal penetration after Li-ESWT treatment without combining PDE5i. This effectiveness of EFD of 0.05mj/mm^2^ of Li-ESWT may be a potentially valid treatment for ED patients, even for the PDE5i nonresponders.

This is the first application of EFD of 0.05mj/mm^2^ of Li-ESWT; we use lower EFD of shockwave but more treated points and more number of shocks. The feasibility and safety have been reported in relevant animal trials. Li et al. ( 16 ) reported that both EFD of 0.06mj/mm^2^ and EFD of 0.09mj/mm^2^ were successful at improving erectile response through cavernous nerve stimulation. They have no significant difference in penile angiogenesis improvement, tissue restoration, and penile nerve regeneration including neuronal nitric oxide synthase (nNOS) positive nerve fibers coinciding with recruitment of endogenous progenitor cells. In a porcine model of skin burn, Goertz et al. ( 17 ) demonstrated that EFD of 0.04mj/mm^2^ of Li-ESWT has a better capacity in angiogenesis and blood flow than other intensity of EFDs. In other similar studies, Lin G and his colleagues investigated the effects of 0.057mj/mm^2^ of EFD and less of it of Li-ESWT in Sprague-Dawley rats. The above scientific research led to the assumption that Li-ESWT also might be beneficial in activating penile progenitor cells in the corpora cavernosa of vasculogenic ED patients ( 18 , 19 ). It is lack in clinical trial about 0.05mj/mm^2^ of Li-ESWT on ED, though the level of EFD has the pathological potential to improve erectile function.

This study is in contrast to trial first reported by Vardi et al. ( 15 ). In their study, 1500 shockwaves were delivered to five treatment points of the penile by a compact electrohydraulic unit (Omnispec ED 1000, USA). Each session comprise 300 shocks on one treatment point at an energy density of 0.09mj/mm^2^ and a frequency of 120/min. It showed that Li-ESWT had a positive short-term clinical physiological effect on erectile function and about 50% of patients got spontaneous erection sufficient for sexual penetration. In our present study, we prefer to 1200 or 1600 shocks were delivered to per treatment point at an energy density of 0.05mj/mm^2^ and a frequency of 200/min, and each session included 10000 shocks and twice a week for 4 week. Our study used shockwave generated electromagnetically and the machine was manufactured in China. The present study and the studies by Vardi are not completely comparable owing to differences in the number of patients, treatment sites, total number of treatments (the Vardi group used 12 treatments) and machines, as explained above.

The mechanism of action that leads to improvement in IIEF-EF scores in men treated with Li-ESWT has not been elucidated completely. The basis for its use is the notion that it could regenerate microvasculature and improving penile hemodynamics. It could induce the release of endothelial or neuronal NOS, vascular endothelial growth factor and proliferating cell nuclear antigen. Studies on the effect of Li-ESWT on penile tissue in rats have shown improvement in erectile function and regeneration of endothelium, smooth muscle, and nerves expressing neuronal nitric oxide synthase ( 19 , 21 ). Studies have shown partial improvement of erectile dysfunction in a diabetic rat model treated with ESWT or stem cells ( 8 , 22 ), and neoangiogenesis in corpora cavernosa in normal rats and diabetic rats treated with ESWT compared with controls ( 8 ).

### Strengths

To our knowledge, this is the first application of 0.05mj/mm^2^ level of EFD of Li-ESWT for ED patients. The effective rate is about 70%, higher than other EFD of Li-ESWT. As expected and akin to published data, 0.05mj/mm^2^ of Li-ESWT appears to be safe and effective in our present study. All of present patients in this study completed the treatment course with no discontinuations and no patient reported penile pain, bruise or others during subsequent follow-up visits.

### Limitations

One limitation of this study is the lack of penile haemodynamic or other objective measurements. We have tested NPT prior to treatment but lack of comparison of change before and after intervention. However, Vardi et al. concluded that NPT is not suitable to be used as an investigative tool due to difficulties in interpreting the results in terms of meaningful parameter changes and changes in penile hemodynamics ( 15 ). Another is that as a result of increasing effect, we could not evaluate how lasting the Li-ESWT benefit was in ED patients. This is the first application of EFD of 0.05mj/mm^2^ of Li-ESWT on ED patients, the number of patients is relatively small. Also the number of patients enrolled in this study was too small to have multivariate analysis. This study did not cover comparisons between different energy densities, such as 0.09mj/mm and 0.05mj/mm. Therefore, the effects of two energy density shock wave treatments cannot be directly compared. Also this is not a controlled study. Further studies with a longer follow-up and a large sample are required to generate a more suitable protocol and optimal level of EFD of Li-ESWT.

## CONCLUSIONS

EFD of 0.05mj/mm^2^of Li-ESWT for ED patients is effective, even part of patients could achieve full erection of EHS of 4. The PDE5i nonresponders became drug-sensitive after EFD of 0.05mj/mm^2^ of Li-ESWT treatment, which meant that after Li-ESWT they were able to achieved a good erection using PDE5i. In PDE5i responders, they were able to have intercourse without medication. Although these are preliminary results, it is expected to be an alternative option for PDE5i nonresponse ED patients for physicians. Additional studies with large sample sizes, longer term studies are required to establish the clinical impact of Li-ESWT.
